# Micro-UltraSound In Cancer – Active Surveillance (MUSIC-AS): A paired, prospective, non-inferiority trial comparing micro-ultrasound and multiparametric MRI at confirmatory biopsy

**DOI:** 10.1016/j.conctc.2026.101639

**Published:** 2026-04-17

**Authors:** Oliver Little, Pat Albers, Tarek A. Bismar, Wayne Brisbane, Stacey Broomfield, Silvia Chang, Christopher Fung, Sunita Ghosh, Tomislav Kuliš, Hugo Lavigueur-Blouin, Giovanni Lughezzani, Miles Mannas, Peter Metcalfe, Luka Penezić, Wendy Tu, Toni Zekulić, Adam Kinnaird

**Affiliations:** a Undergraduate Medical Education, Cumming School of Medicine, University of Calgary, Calgary, Canada; b Division of Urology, Department of Surgery, University of Alberta, Edmonton, Canada; c Department of Pathology & Laboratory Medicine, Oncology and Biochemistry and Molecular Biology, University of Calgary, Calgary, Canada; d Alberta Prostate Cancer Research Initiative (APCaRI), Edmonton, Canada; e Institute of Urologic Oncology, Department of Urology, UCLA Medical Center, University of California Los Angeles, USA; f Department of Urology, University of Florida, Gainesville, USA; g Alberta Centre for Urologic Research and Excellence, Edmonton, Canada; h Department of Radiology, University of British Columbia, Vancouver, Canada; i Department of Radiology & Diagnostic Imaging, University of Alberta, Edmonton, Canada; j Department of Oncology, University of Alberta, Edmonton, Canada; k Department of Urology, University Hospital Center Zagreb, Zagreb, Croatia; l Division of Urology, Department of Surgery, Université de Montréal, Quebec, Canada; m Departments of Urology and Biomedical Sciences, Humanitas Clinical and Research Center, Rozzano, Italy; n Department of Biomedical Sciences, Humanitas University, Pieve Emanuele, Italy; o Vancouver Prostate Centre, Mohseni Institute of Urologic Sciences, Vancouver, British Columbia, Canada; p Cancer Research Institute of Northern Alberta, Edmonton, Canada

**Keywords:** Prostate cancer, Active surveillance, Prostate biopsy, Targeted biopsy, Micro-ultrasound, Multiparametric MRI, Non-inferiority trial

## Abstract

**Background:**

Accurate risk assessment is essential for men on active surveillance (AS) of prostate cancer (PCa). Multiparametric MRI improves detection of clinically significant PCa (csPCa; Grade Group ≥2) but is limited by registration error, contraindications and long wait times. Micro-ultrasound (microUS) is a high-resolution, real-time imaging modality that has been shown to be non-inferior to MRI for the detection of csPCa in biopsy-naïve men. However, trials comparing microUS against MRI in men managed by AS remain limited.

**Methods:**

MUSIC-AS is a paired, within-subject, prospective non-inferiority diagnostic trial comparing microUS and MRI for the detection of csPCa in men undergoing confirmatory biopsy during AS. Eligible participants include men with previously diagnosed Grade Group 1 PCa requiring confirmatory biopsy. Each participant will undergo pre-biopsy MRI followed by a microUS/MRI-guided biopsy (in which the operator is initially blinded to the MRI during acquisition of microUS targeted cores) followed by MRI targeted cores and systematic sampling. The primary endpoint is the detection of csPCa by combined targeted plus systematic biopsies. The planned sample size is 210 participants.

**Results:**

The primary hypothesis is non-inferiority of the csPCa detection rate between microUS-targeted plus systematic biopsies and MRI-targeted plus systematic biopsies. Secondary analyses will evaluate upgrade-free probabilities at 2, 5, and 10 years after confirmatory biopsy. An exploratory analysis using germline sequencing will be performed.

**Conclusions:**

MUSIC-AS will determine whether micro-ultrasound is non-inferior to MRI for detecting csPCa in men on active surveillance. Confirmation of non-inferiority may support microUS as a cost-effective, time-efficient, and widely accessible imaging alternative in this population.

## 1 Background & rationale

Prostate cancer (PCa) is the most common internal malignancy in men, and its management has changed considerably over the last 20 years [1]. Active surveillance (AS) has emerged as a standard-of-care option selected by >40% of men with low-risk PCa [[1], [2], [3], [4]]. AS limits risks to sexual, urinary, and bowel function compared to surgery or radiation [5]. The decision to enter AS rather than undergo treatment is primarily based on tumour aggressiveness, making accurate assessment of this staging key to avoid missing high-risk tumours that can become lethal if not treated. In most AS programs, men enter based on a random, non-targeted biopsy revealing a low-risk cancer with Gleason Score of 6 (ISUP Grade Group 1, GG1) and undergo periodic biopsies to confirm that this low-risk categorization is maintained. However, many men (∼35% at 5 years) will exhibit higher risk PCa that requires active treatment [2,3].

Several new technologies have been developed to improve the accuracy of this risk stratification, including advanced imaging (multiparametric magnetic resonance imaging [MRI] and micro-ultrasound [microUS]), targeted prostate biopsy, and germline sequencing (to determine hereditary predisposition to aggressive cancer phenotypes).

### 1.1 Multiparametric MRI

MRI has become an increasingly important non-invasive element of the PCa diagnostic pathway, enabling enhanced risk stratification and tumor identification compared to traditional US-based approaches [6]. MRI is scored using the Prostate Imaging-Reporting and Data System (PI-RADS), with scores of 1 and 2 considered low-risk and 3-5 suspicious for PCa [7]. Areas of suspicion, called regions of interest (ROIs), are targeted by the clinician, either cognitively or with the use of fusion software.

The incorporation of MRI into the diagnostic workflow improves detection of aggressive PCa [8]. MRI detects ∼90% of Grade Group 3 PCa and 75% of Grade Group 2 PCa [9,10]. However, as MRI is performed at a separate time than prostate biopsy, this may lead to registration errors caused by organ movement or other factors. MRI is contraindicated in men with chronic kidney disease, claustrophobia, and ferromagnetic implants, and resolution is limited in men with previous hip replacements [11].

### 1.2 Micro-ultrasound

A novel 29 MHz high-resolution microUS device (called ExactVu™) has been specifically manufactured for prostate imaging and biopsy. MicroUS has an imaging resolution of 70 μm (0.07 mm), providing a 300% improvement on standard ultrasound [12]. The increased resolution of microUS allows real-time visualization of PCa, negating registration error. The 70-μm resolution is equivalent to the diameter of a typical prostatic duct; as PCa malignancy alters the prostate ductal architecture, the microUS is able to visualize these changes. MicroUS is scored using the Prostate Risk Identification using MicroUltraSound (PRI-MUS) system, with scores of 1 and 2 considered low-risk and 3-5 suspicious for PCa [13]. The OPTIMUM randomized controlled trial provided level 1 evidence that microUS is non-inferior to MRI for the detection of Gleason Grade Group 2 or higher PCa in biopsy naïve men [14]. The only contraindication to microUS is men lacking a rectum (e.g., previously surgically removed) or other physical constraint (e.g., anal stricture) preventing rectal insertion of the ultrasound probe.

### 1.3 Targeted prostate biopsy

New biopsy techniques, called targeted prostate biopsies, incorporate advanced image-fusing software into the biopsy procedure. One common form of targeted biopsy is called MRI/US fusion biopsy. MRI/US fusion biopsy uses software to fuse the MRI identified ROIs images with the real-time sonogram during the biopsy procedure. Targeted biopsy detects more aggressive PCa and results in fewer upgrading events at final pathology after surgical removal than non-targeted ultrasound-guided biopsy (3.5% vs 30%) [6,8,15]. Use of targeted biopsy may better determine risk of harbouring aggressive PCa in men before entry and during AS [[16], [17], [18], [19], [20], [21], [22]]. Short-term results (2 years of follow-up) show that MRI/US fusion biopsy at the initiation of AS reduced further PCa upgrading by 50% compared with non-targeted ultrasound-guided biopsy [23]. Similarly, MRI/microUS fusion is available using the ExactVu™ device.

### 1.4 Rationale

This trial aims to use and compare two advanced imaging technologies (MRI and microUS) to determine if microUS is non-inferior to MRI in detection of GG2 or higher PCa at biopsy during AS. This trial will provide evidence that may be used to reduce unnecessary biopsies, decrease costs and reduce wait times, as well as reduce expenditures and ensure that aggressive tumours are caught early and proceed to curative treatment.

## 2 Design & methods

This multicenter trial will enrol men internationally, capturing a diverse population and creating generalizability. All microUS operators will be required to have ‘Advanced Users’ status from Exact Imaging.

### 2.1 Prospective paired diagnostic non-inferiority trial

This multicenter trial is a prospective, paired, diagnostic, non-inferiority trial to compare the detection of ≥GG2 PCa by MRI versus microUS for men on AS for PCa (men previously diagnosed with GG1, requiring a confirmatory biopsy). All patients will undergo a prebiopsy MRI, to which the clinician performing the biopsy will be initially blinded. Either transrectal or transperineal biopsy approaches may be used for this trial.

### 2.2 Biopsy procedure

**Fig. 1:**
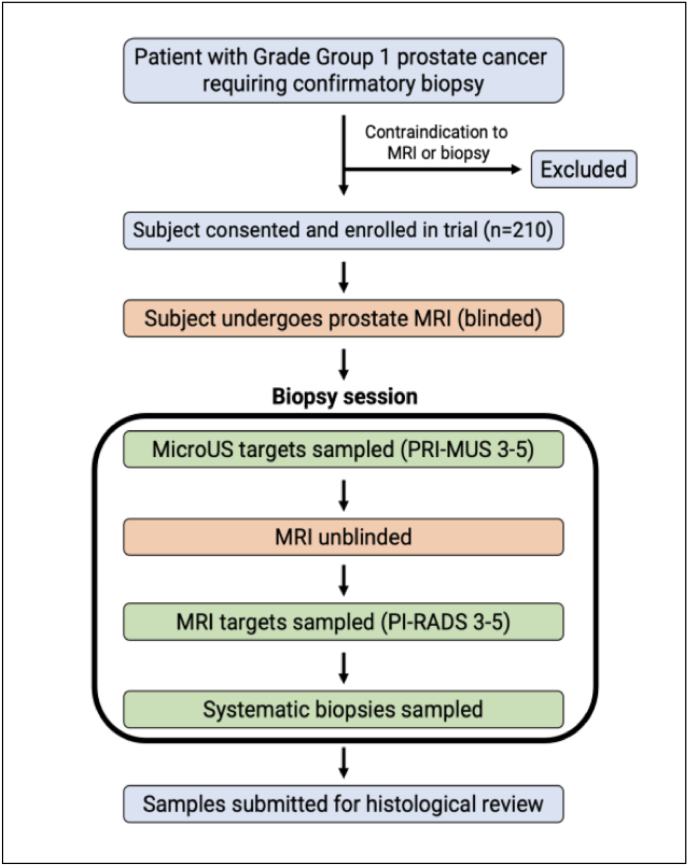
Biopsy schematic for MUSIC-AS.

The biopsy procedure will be conducted as follows (Fig. 1):

I. A prebiopsy MRI of the prostate will be obtained in a manner by which the clinician performing the biopsy will remain blinded to MRI results.
II. While blinded, microUS-guided biopsies of all PRI-MUS 3-5 ROIs will be performed.
III. Following completion of microUS sampling, MRI/microUS fusion software will be activated and the clinician unblinded to the MRI results. Biopsy cores will then be obtained from any PI-RADS 3–5 ROIs identified on MRI, using the fusion platform.
  a. Up to two ROIs with the highest PRI-MUS or PI-RADS scores will be targeted with up to three cores per ROI.
  b. If MRI and microUS ROIs overlap, the previously obtained microUS cores will be considered representative of both imaging modalities.
IV. After targeted sampling, a standard 12-core systematic biopsy will be performed.
V. All collected cores will be submitted for histological review.

### 2.3 Patient population

Eligible participants will be identified and potentially recruited if they meet the inclusion criteria outlined in Table 1.

**Table 1:** Eligibility criteria.

Category	Criteria
Inclusion Criteria	• Adult men with Gleason Grade Group 1 PCa managed by active surveillance who require a confirmatory prostate biopsy. • Adult men who are able to undergo prostate MRI.
Exclusion Criteria	• Unable to provide informed consent. Contraindication to prostate MRI (e.g., severe claustrophobia, ferromagnetic implant, renal impairment). Contraindication to prostate biopsy (e.g., uncorrectable coagulopathy, active infection).

### 2.4 Outcomes

#### 2.4.1 Primary

The primary outcome will be the csPCa (defined as Grade Group ≥2) rate between microUS-targeted plus systematic biopsies and MRI-targeted plus systematic biopsies. The detection rate is defined as the proportion of men in whom csPCa (Grade Group ≥2) is identified at confirmatory biopsy.

#### 2.4.2 Secondary: Comparative performance of image guided and systematic biopsies

The relative detection rate of csPCa between each imaging-guided biopsy technique and the concurrent standard 12-core systematic biopsy performed during the same session.

#### 2.4.3 Secondary: Long-term upgrading

Time to histologic upgrading, defined as progression from Grade Group 1 to ≥ Grade Group 2 disease, on subsequent follow-up biopsies during active surveillance and upgrade-free probabilities at 2, 5, and 10 years after confirmatory biopsy.

#### 2.4.4 Exploratory: Genomic correlation

Association between biopsy findings and germline genetic variants identified from sequencing of blood samples collected at time of confirmatory biopsy.

#### 2.4.5 Exploratory: Lesion location

Exploratory analyses will also examine csPCa lesion location, including peripheral versus anterior zone prostatic disease, and compare the distribution of csPCa detected by microUS-targeted and MRI-targeted biopsy.

### 2.5 Statistical considerations

#### 2.5.1 Primary – microUS vs. MRI

This study is a paired, prospective, non-inferiority diagnostic trial comparing the detection rate of csPCa (Grade Group ≥2) between microUS and mpMRI during confirmatory biopsy in men on active surveillance. Each participant undergoes both MRI-guided and microUS-guided biopsies in the same session; thus, analyses account for within-subject pairing to control for inter-individual variability. Previous confirmatory biopsy data demonstrate comparable detection rates between MRI (32%) and micro-ultrasound (31%) for csPCa (≥ Grade Group 2) [24,25]. These findings support the non-inferiority framework of the present study. Based on consensus within the study team and a previously published Level 1 evidence trial comparing microUS to MRI [14], a non-inferiority threshold of 10% will be used. Using a one-sided non-inferiority test of the difference between two correlated proportions, a sample of 183 subjects achieves 90% power with a significance level of 0.05 when the non-inferiority margin is −0.10. Sample size was calculated using PASS version 19 software and non-inferiority tests for the difference between two correlated proportions was used. We plan to recruit 200 patients and added 5% to account for dropout or incomplete data, with our planned recruitment number set at 210.

#### 2.5.2 Secondary – Comparative performance

Detection rates for each imaging-guided biopsy technique will be compared to the standard 12-core systematic biopsy obtained during the same session using the same analytic approach as for the primary endpoint. Paired-proportion analyses (McNemar's test) will assess whether either imaging-guided technique detects a significantly different proportion of csPCa compared to the systematic biopsy.

#### 2.5.3 Secondary – Time to upgrading

Time-to-event outcomes, defined as progression from GG1 to ≥ GG2, will be analyzed using Kaplan–Meier survival analysis. Median upgrade-free survival and 2-, 5-, and 10-year upgrade-free probabilities will be reported. Censoring will occur at the date of definitive therapy, withdrawal, or last follow-up biopsy. Cox proportional-hazards regression will estimate hazard ratios for upgrading, adjusting for predefined baseline variables (age, PSA, PSA density, PRI-MUS and PI-RADS score, number of positive cores).

## 3 Discussion and limitations

There have been two limitations identified in this study, which we have tried to address in our design. First is the inclusion of Grade Group 2 as csPCa for the primary outcome. There is controversy as to whether this is indeed clinically significant, or if these cancers could continue to be monitored by AS. The inclusion of GG2 as csPCa for the primary outcome warrants consideration. Although GG2 is commonly used as the threshold for clinically significant disease, and as such for discontinuation of active surveillance, there is some evidence that suggest men with favourable intermediate risk GG2 PCa may have acceptable long-term oncologic outcomes on AS. For the purposes of this trial, we justify ≥ GG2 disease as the endpoint for csPCa as Gleason Grade Group 2 is the threshold histological grade at which radical therapy is indicated, as well as comparison to many long-term AS studies that use grade reclassification to Grade Group 2 or more as a trigger to discontinue AS. In short, GG2 remains the most practical and literature-consistent threshold for upgrade to clinically significant prostate cancer; however, we acknowledge that not all GG2 disease carries equivalent risk. Second, it is possible that biopsy operators are inadvertently made aware of the MRI results prior to the official unblinding period. This can be assessed as all images (deidentified) are uploaded to a central imaging repository and we are able to determine at which point the MRI/microUS fusion software was activated. This method was performed for the OPTIMUM trial with the finding that there was no early unblinding for any patient in the trial.

Another emerging tool in prostate cancer detection is PSMA PET/CT. Early data demonstrate that PSMA PET/CT may identify suspicious prostatic lesions in men on AS and may play a role complementing MRI in certain settings. Evidence remains limited however, and current data are insufficient to support PSMA PET/CT as a replacement for established surveillance strategies for men on AS for their PCa. Further studies may help define whether PSMA PET/CT has a role as an adjunct modality in the detection of csPCa.

Genetic evaluation may also become increasingly relevant for men with GG1 PCa considered for or managed with AS. In particular, germline alterations associated with aggressive prostate cancer phenotypes, including BRCA1/2 gene mutations, may refine baseline risk assessment, support germline testing, and inform family counseling. For our trial, the planned exploratory germline sequencing component may provide preliminary data on the relationship between inherited risk variants and upgrading at confirmatory biopsy.

## 4 Conclusion

The MUSIC-AS trial will determine whether confirmatory biopsy using microUS is non-inferior to biopsy using MRI. If demonstrated to be non-inferior this trial will support microUS as a cost-effective, time-efficient, and potentially widely accessible imaging alternative for men managed by AS.

## CRediT authorship contribution statement

**Oliver Little:** Writing – review & editing, Writing – original draft. **Pat Albers:** Writing – original draft.

## Funding

This work was supported by the Kaye Edmonton Clinic Grant, University Hospital Foundation.

## Declaration of competing interest

The authors declare the following financial interests/personal relationships which may be considered as potential competing interests: Dr Adam Kinnaird reports financial support was provided by Kaye Edmonton Clinic, University Hospital Foundation. Additionally, Dr Wayne Brisbane and Dr Adam Kinnaird have received funding from Exact Imaging for research. The remaining authors declare no known competing financial interests or personal relationships that could have appeared to influence the work reported in this paper.

## Data Availability

No data was used for the research described in the article.
